# Supplementary material for: Plasma biomarkers predict Alzheimer’s disease before clinical onset in Chinese cohorts
Source: Nat Commun. 2023 Oct 24;14:6747. doi: 10.1038/s41467-023-42596-6 (PMC10597998; doi:10.1038/s41467-023-42596-6)
Supplement: Supplementary file 1 — Supplementary Information [file 41467_2023_42596_MOESM1_ESM.pdf]

Supplementary Information

Plasma Biomarkers Predict Alzheimer's Disease Before Clinical Onset in Chinese Cohorts

Cai *et al.*

Supplementary Table 1. Plasma Biomarker Levels of Participants in Cohort 1.

Supplementary Table 2. Details of Diagnostic Models of Plasma Biomarkers.

Supplementary Table 3. Information on Simoa for Plasma Biomarkers.

Supplementary Table 4. Information on ELISA Kits for CSF Biomarkers.

Supplementary Fig.1. Prediction of Plasma Aβ42, P-tau181, and NfL for Different FAD Mutation Carriers in Cohort 2.

Supplementary Fig.2. Cutoff Values of CSF Biomarker to Determine AD in Cohort 1.

Supplementary discussion

Supplementary references

Supplementary Table 1. Plasma Biomarker Levels of Participants in Cohort 1.

Biomarkers	Baseline	Follow-up	*P* value
Aβ42	15.50(4.09)	12.43(4.10)	2.50×10^{-54}
Aβ40	212.76(86.63)	194.23(64.82)	2.64×10^{-8}
t-tau	1.92(0.71)	2.17(0.71)	1.59×10^{-18}
p-tau181	2.14(0.99)	3.02(1.48)	2.58×10^{-36}
NfL	12.04(4.67)	14.18(4.85)	4.58×10^{-24}

All variables are presented as "mean (standard deviation)" in the table. All biomarker values are provided in terms of pg/mL. *P* values were derived from two-sided paired t-tests without adjustment for multiple comparisons.

Abbreviations: Aβ, amyloid-β; NfL: neurofilament light chain; p-tau, phosphorylated tau; t-tau, total tau.

Supplementary Table 2. Details of Diagnostic Models of Plasma Biomarkers.

Model	Odds ratio (95% CI)	*P* value (Logistic model)[a]	AIC	AUC (95% CI)	*P* value (AUC comparison)[b]
Cohort 1<br>Baseline model 1<br>Plasma Aβ42	 <br>0.86 (0.80-0.92)	 <br>1.37×10^{-5}	 327.57	 0.66 (0.59-0.72)	 9.95×10^{-6}
Cohort 1<br>Baseline model 2<br>Plasma p-tau181	 <br>1.77 (1.33-2.36)	 <br>8.16×10^{-5}	 331.63	 0.64 (0.57-0.71)	 1.95×10^{-6}
Cohort 1<br>Baseline model 3<br>Plasma NfL	 <br>1.15 (1.08-1.22)	 <br>2.42×10^{-5}	 328.27	 0.67 (0.60-0.73)	 1.24×10^{-5}
Cohort 1<br>Baseline model 4<br>Plasma Aβ42<br>Plasma p-tau181<br>Plasma NfL	 <br>0.84 (0.78-0.91)<br>1.98 (1.45-2.70)<br>1.16 (1.08-1.25)	 <br>1.39×10^{-5}<br>1.66×10^{-5}<br>2.70×10^{-5}	 291.47	 0.78 (0.71-0.83)	 3.37×10^{-2}
Cohort 1<br>Baseline model 5<br>*APOE*<br>Plasma Aβ42<br>Plasma p-tau181<br>Plasma NfL	 <br>4.06 (2.07-7.97)<br>0.85 (0.78-0.92)<br>2.12 (1.53-2.93)<br>1.18 (1.10-1.27)	 <br>4.43×10^{-5}<br>6.40×10^{-5}<br>6.84×10^{-6}<br>7.15×10^{-6}	 275.31	 0.81 (0.75-0.86)	 /
Cohort 1<br>Follow-up model 1<br>Plasma Aβ42	 <br>0.46 (0.38-0.56)	 <br>4.05×10^{-15}	 176.24	 0.92 (0.89-0.96)	 7.13×10^{-6}
Cohort 1<br>Follow-up model 2<br>Plasma p-tau181	 <br>5.49 (3.63-8.29)	 <br>7.11×10^{-16}	 205.83	 0.89 (0.86-0.93)	 2.60×10^{-7}
Cohort 1<br>Follow-up model 3<br>Plasma NfL	 <br>1.29 (1.20-1.39)	 <br>4.19×10^{-11}	 287.21	 0.77 (0.71-0.83)	 4.32×10^{-14}
Cohort 1<br>Follow-up model 4<br>Plasma Aβ42<br>Plasma p-tau181<br>Plasma NfL	 <br>0.39 (0.27-0.54)<br>9.74 (4.06-23.35)<br>1.29 (1.09-1.52)	 <br>3.18×10^{-8}<br>3.33×10^{-7}<br>2.86×10^{-3}	 82.27	 0.99 (0.98-1.00)	 0.17
Cohort 1<br>Follow-up model 5<br>*APOE*<br>Plasma Aβ42<br>Plasma p-tau181<br>Plasma NfL	 <br>3.96 (1.05-14.85)<br>0.38 (0.27-0.54)<br>10.14 (4.16-24.71)<br>1.32 (1.10-1.58)	 <br>4.15×10^{-2}<br>1.18×10^{-7}<br>3.49×10^{-7}<br>2.24×10^{-3}	 79.87	 0.99 (0.98-1.00)	 /
Cohort 2					

Model 1<br>Plasma Aβ42	 0.86 (0.78-0.95)	 1.86×10^{-3}	135.65	0.68 (0.57-0.78)	1.40×10^{-2}
Cohort 2<br>Model 2<br>Plasma p-tau181	 <br>3.31 (1.68-6.52)	 <br>5.26×10^{-4}	132.51	0.71 (0.61-0.81)	4.15×10^{-2}
Cohort 2<br>Model 3<br>Plasma NfL	 <br>1.23 (1.08-1.41)	 <br>2.31×10^{-3}	135.92	0.69 (0.59-0.80)	3.42×10^{-2}
Cohort 2<br>Model 4<br>Plasma Aβ42<br>Plasma p-tau181<br>Plasma NfL	 <br>0.87 (0.78-0.96)<br>2.98 (1.39-5.95)<br>1.18 (1.03-1.35)	 <br>6.80×10^{-3}<br>4.38×10^{-3}<br>1.80×10^{-2}	120.93	0.79 (0.71-0.88)	/

[a] Statistical analyses were two-sided without adjustment for multiple comparisons.

[b] Uncorrected two-sided *P* values from DeLong test comparing AUCs of baseline model 1, 2, 3 and 4 with baseline model 5 in cohort 1, follow-up model 1, 2, 3 and 4 with follow-up model 5 in cohort 1, and model 1, 2, and 3 with model 4 in cohort 2.

Abbreviations: Aβ, amyloid-β; AIC: Akaike information criterion; *APOE,* apolipoprotein E; AUC: areas under the curve; CI: confidence interval; NfL: neurofilament light chain; p-tau, phosphorylated tau.

Supplementary Table 3. Information on Simoa for Plasma Biomarkers.

Simoa kits	Catalog numbers	Assay ranges (pg/ml)	Current data ranges (pg/ml)[a]
**Aβ42**			
Quanterix (USA)	101995	0-240	3.51-27.76
**Aβ40**			
Quanterix (USA)	101995	0-560	43.0-522.26
**t-tau**			
Quanterix (USA)	101995	0-400	0.43-4.97
**p-tau181**			
Quanterix (USA)	103714	0-424	0.49-7.48
**NfL**			
Quanterix (USA)	103186	0-1800	4.25-33.55

[a] The current data ranges indicate the lowest and highest values obtained by using corresponding Simoa kits.

Abbreviations: Aβ, amyloid-β; NfL, neurofilament light chain; p-tau, phosphorylated tau; t-tau, total tau.

Supplementary Table 4. Information on ELISA Kits for CSF Biomarkers.

ELISA kits	Catalog numbers	Assay ranges (pg/ml)	Current data ranges (pg/ml)[a]
**Aβ42**			
INNOTEST (Japan)	81576	62.5-4000	129-1109
**Aβ40**			
INNOTEST (Japan)	81585	7.8-1000	3231-21899[b]
**t-tau**			
INNOTEST (Japan)	81572	34-2500	81-1141
**p-tau181**			
INNOTEST (Japan)	81581	15.6-1000	15.7-276
**NfL**			
INNOTEST (Japan)	20-8002	0.5-40	513-2915[b]

[a] The current data ranges indicate the lowest and highest values obtained by using corresponding ELISA kits.

[b] indicates that proteins were diluted according to the pilot study data and the assay range of the ELISA kit. Other values were in the assay ranges when using the ELISA kits.

Abbreviations: Aβ, amyloid-β; ELISA: enzyme-linked immunosorbent assay; NfL, neurofilament light chain; p-tau, phosphorylated tau; t-tau, total tau.

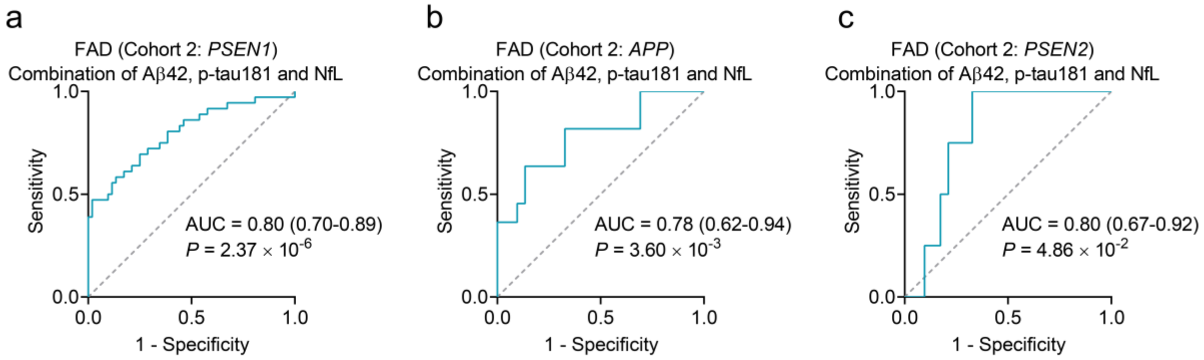

Supplementary Fig.1. Prediction of Plasma Aβ42, P-tau181, and NfL for Different FAD Mutation Carriers in Cohort 2.

Receiver operating characteristic curve analysis and corresponding AUCs for differentiating *PSEN1* (a), *APP* (b), and *PSEN2*(c) mutation carriers from non-carriers. Models were generated from a combination of plasma Aβ42, p-tau181, and NfL. All analyses were two-sided without adjustment for multiple comparisons. n = 52 (control), 36 (*PSEN1* mutation carriers), 11 (*APP* mutation carriers), 4 (*PSEN2* mutation carriers). Aβ, amyloid-β; *APP*: amyloid precursor protein; AUC: area under the curve; FAD, familial Alzheimer’s disease; NfL, neurofilament light chain; Mut, mutation carrier; *PSEN*: presenilin; p-tau, phosphorylated tau. Source data are provided as a Source Data file.

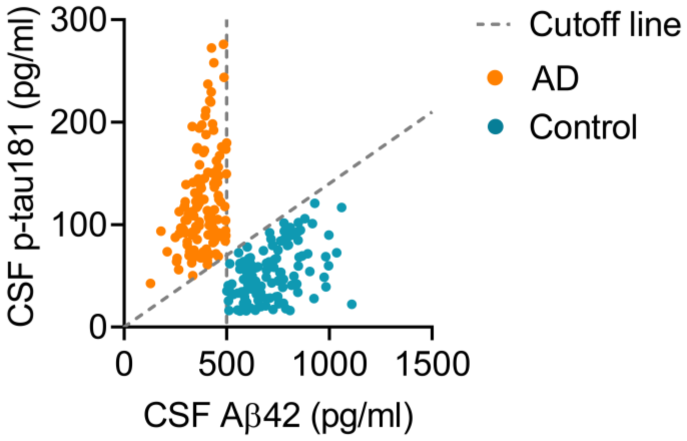

Supplementary Fig.2. Cutoff Values of CSF Biomarker to Determine AD in Cohort 1.
The cutoff values of CSF p-tau181/ Aβ42 (0.14) and Aβ42 (500pg/mL) were used to determine AD in cohort 1. Blue dots indicate controls while orange dots indicate participants with AD. The dotted line (slope = 0.14) indicates the cutoff for p-tau181/Aβ42; 500 pg/ml in the horizontal axis indicates the cutoff value of Aβ42. n = 123 (control), 126 (AD). Aβ, amyloid-β; AD, Alzheimer's disease; CSF, cerebrospinal fluid; p-tau, phosphorylated tau. Source data are provided as a Source Data file.

Supplementary discussion

According to the literature in this field, although it has been shown that about half of the cerebrospinal fluid (CSF) proteins are moderately related to their plasma counterparts[1], the correlations between plasma and CSF biomarkers are not exactly the same in previous studies. For instance, in one study[2] on phosphorylated tau (p-tau)181, the β coefficient was 0.73 in the correlation analyses between plasma and CSF p-tau181, which is completely consistent with our data (β=0.73, correlation between plasma and CSF p-tau181), while the values of r in another study[3] were 0.7055 (plasma vs. CSF) and 0.7937 (serum vs. CSF), which were higher or lower than ours (our r=0.73). In other studies[4–8], r values ranged between 0.30–0.65, indicating that the degree of correlation between plasma and CSF p-tau181 was not consistent across studies. A similar situation exists in the correlation between plasma and CSF neurofilament light chain (NfL); for instance, some studies[9,10] (r=0.72 or 0.78) showed r values similar to ours (our r=0.77), some (r =0.86)[7] higher than ours, and some (r =0.64 or 0.59)[11,12] lower than ours. For amyloid-β (Aβ)42, our results were higher than those of previous studies (our r=0.81 vs. others r=0.35–0.41)[13,14]. We speculate that this may be partly due to our strict inclusion criteria of Alzheimer's disease (AD) involving CSF biomarkers (p-tau181/Aβ42>0.14 and Aβ42<500 pg/mL). Additionally, results of studies on the correlation between plasma markers and CSF were quite different, even in the same article. For example, one study[2] showed that a correlation analysis of p-tau181 was β=0.73 in dataset one, and β=0.52 in dataset two, while another study[15] showed the results for NfL, R^2=0.27 in BioFINDER-1 vs. R^2=0.49 in BioFINDER-2, for glial fibrillary acidic protein, R^2=0.13 in BioFINDER-1 vs. R^2=0.43 in BioFINDER-2. The above data indicate that in the correlation analysis between plasma and CSF, the correlation strength of biomarkers varies among studies or even in different datasets of the same study. The underlying reasons for the inconsistency remain unclear and could be attributed to methodological

variations, heterogeneity in the selected cohort, or the use of specific inclusion criteria (i.e. CSF biomarkers vs. positron emission tomography). However, it also suggests that these plasma biomarkers can partially reflect their counterpart levels in CSF, which to some extent enhances confidence in using plasma biomarkers to aid the diagnosis of AD.

Supplementary references

1. Whelan, C. D. *et al.* Multiplex proteomics identifies novel CSF and plasma biomarkers of early Alzheimer's disease. *Acta Neuropathol. Commun.* **7**, 169 (2019).
2. Janelidze, S. *et al.* Plasma P-tau181 in Alzheimer's disease: relationship to other biomarkers, differential diagnosis, neuropathology and longitudinal progression to Alzheimer's dementia. *Nat. Med.* **26**, 379–386 (2020).
3. Karikari, T. K. *et al.* Blood phosphorylated tau 181 as a biomarker for Alzheimer's disease: a diagnostic performance and prediction modelling study using data from four prospective cohorts. *Lancet Neurol.* **19**, 422–433 (2020).
4. Altomare, D. *et al.* Plasma biomarkers for Alzheimer's disease: a field-test in a memory clinic. *J. Neurol. Neurosurg. Psychiatry* **94**, 420–427 (2023).
5. Tropea, T. F. *et al.* Plasma phosphorylated tau181 predicts cognitive and functional decline. *Ann. Clin. Transl. Neurol.* **10**, 18–31 (2023).
6. Karikari, T. K. *et al.* Diagnostic performance and prediction of clinical progression of plasma phospho-tau181 in the Alzheimer's Disease Neuroimaging Initiative. *Mol. Psychiatry* **26**, 429–442 (2021).
7. Álvarez-Sánchez, L. *et al.* Assessment of plasma and cerebrospinal fluid biomarkers in different stages of Alzheimer's disease and frontotemporal dementia. *Int. J. Mol. Sci.* **24**, 1226 (2023).

8. Palmqvist, S. *et al.* An accurate fully automated panel of plasma biomarkers for Alzheimer's disease. *Alzheimers Dement.* **19**, 1204–1215 (2023).
9. Alagaratnam, J. *et al.* Correlation between CSF and blood neurofilament light chain protein: a systematic review and meta-analysis. *BMJ Neurol. Open* **3**, e000143 (2021).
10. Alcolea, D. *et al.* Use of plasma biomarkers for AT(N) classification of neurodegenerative dementias. *J. Neurol. Neurosurg. Psychiatry* **92**, 1206–1214 (2021).
11. Parvizi, T. *et al.* Real-world applicability of glial fibrillary acidic protein and neurofilament light chain in Alzheimer's disease. *Front. Aging Neurosci.* **14**, 887498 (2022).
12. Mattsson, N., Andreasson, U., Zetterberg, H., Blennow, K., & Alzheimer's Disease Neuroimaging Initiative. Association of plasma neurofilament light with neurodegeneration in patients with Alzheimer disease. *JAMA Neurol.* **74**, 557 (2017).
13. Nakamura, A. *et al.* High performance plasma amyloid-β biomarkers for Alzheimer's disease. *Nature* **554**, 249–254 (2018).
14. Klafki, H.-W. *et al.* Diagnostic performance of automated plasma amyloid-β assays combined with pre-analytical immunoprecipitation. *Alzheimers Res. Ther.* **14**, 127 (2022).
15. Pichet Binette, A. *et al.* Confounding factors of Alzheimer's disease plasma biomarkers and their impact on clinical performance. *Alzheimers Dement.* **19**, 1403–1414 (2023).
