# Supplementary material for: Plasma biomarkers predict Alzheimer’s disease before clinical onset in Chinese cohorts
Source: Nat Commun. 2023 Oct 24;14:6747. doi: 10.1038/s41467-023-42596-6 (PMC10597998; doi:10.1038/s41467-023-42596-6)
Supplement: Supplementary file 3 — Reporting Summary [file 41467_2023_42596_MOESM3_ESM.pdf]

nature portfolio

Corresponding author(s): Longfei Jia

Last updated by author(s): Oct 9, 2023

Reporting Summary

Nature Portfolio wishes to improve the reproducibility of the work that we publish. This form provides structure for consistency and transparency in reporting. For further information on Nature Portfolio policies, see our Editorial Policies and the Editorial Policy Checklist.

Statistics

For all statistical analyses, confirm that the following items are present in the figure legend, table legend, main text, or Methods section.

n/a	Confirmed	
☐	☒	The exact sample size (n) for each experimental group/condition, given as a discrete number and unit of measurement
☐	☒	A statement on whether measurements were taken from distinct samples or whether the same sample was measured repeatedly
☐	☒	The statistical test(s) used AND whether they are one- or two-sided<br>*Only common tests should be described solely by name; describe more complex techniques in the Methods section.*
☐	☒	A description of all covariates tested
☐	☒	A description of any assumptions or corrections, such as tests of normality and adjustment for multiple comparisons
☐	☒	A full description of the statistical parameters including central tendency (e.g. means) or other basic estimates (e.g. regression coefficient) AND variation (e.g. standard deviation) or associated estimates of uncertainty (e.g. confidence intervals)
☐	☒	For null hypothesis testing, the test statistic (e.g. F, t, r) with confidence intervals, effect sizes, degrees of freedom and P value noted<br>*Give P values as exact values whenever suitable.*
☒	☐	For Bayesian analysis, information on the choice of priors and Markov chain Monte Carlo settings
☒	☐	For hierarchical and complex designs, identification of the appropriate level for tests and full reporting of outcomes
☐	☒	Estimates of effect sizes (e.g. Cohen's d, Pearson's r), indicating how they were calculated

Our web collection on statistics for biologists contains articles on many of the points above.

Software and code

Policy information about availability of computer code

Data collection	No software was used for data collection.
Data analysis	The data analyses were performed with SPSS v.22,R studio and R version 4.2.3. Packages used are: DescTools 0.99.48, pROC 1.18.0 and ggplot2 3.3.6. The code used for producing the results presented in this study is available at Zenodo: https://zenodo.org/record/837570 with a DOI:10.5281/ZENODO.8375702.

For manuscripts utilizing custom algorithms or software that are central to the research but not yet described in published literature, software must be made available to editors and reviewers. We strongly encourage code deposition in a community repository (e.g. GitHub). See the Nature Portfolio guidelines for submitting code & software for further information.

Data

Policy information about availability of data

All manuscripts must include a data availability statement. This statement should provide the following information, where applicable:

- Accession codes, unique identifiers, or web links for publicly available datasets
- A description of any restrictions on data availability
- For clinical datasets or third party data, please ensure that the statement adheres to our policy

The de-identified raw data are available from the corresponding author Longfei Jia (longfei@mail.ccmu.edu.cn) to researchers who provide methodologically sound scientific proposals. A materials transfer and/or data access agreement will be required for accessing shared data. Source data are provided with this paper.

Research involving human participants, their data, or biological material

Policy information about studies with human participants or human data. See also policy information about sex, gender (identity/presentation), and sexual orientation and race, ethnicity and racism.

Reporting on sex and gender	Sex was self-reported. This is sex-matched study with the male to female ratio of 49.8 to 50.2 in cohort 1 and 48.5 to 51.5 in cohort 2. Sex was considered as a covariate and incorporated into models. No sex-specific comparison analysis was performed.
Reporting on race, ethnicity, or other socially relevant groupings	Not applicable.
Population characteristics	All this information is given in Tables 1 and 2.
Recruitment	Cohort 1 consisted of participants from the China Cognition and Aging Study who were cognitively intact at baseline 8 to 10 years before the study (between 2012 and 2014). Blood samples were collected at baseline and follow-up; CSF samples were collected only at follow-up. Biomarkers in both blood and CSF were measured at follow-up. The diagnosis of AD was based on both the clinical and biomarker criteria. Clinical diagnosis of AD was established according to the 2011 NIA-AA criteria and must meet a biomarker criterion (P-tau181/Aβ42 > 0.14) based on our previously published data. Furthermore, we used a reported CSF Aβ42 value of < 500 pg/ml as another inclusion criterion because low CSF Aβ42 is a critical pathological change in AD according to the ATN framework. In cohort 1, participants who were cognitively intact at baseline (8-10 years earlier) but later developed AD were determined to have preclinical AD at baseline. The onset of the disease refers to cognitive impairment reaching the diagnostic criteria of AD, while cognitive impairment reaching the diagnostic criteria of MCI was not counted as onset until cognitive decline meets the diagnostic criteria of AD. Participants who remained cognitively intact during the follow-up were considered normal controls.<br>The replication cohort (Cohort 2) was recruited from the Chinese Familial Alzheimer's Disease Network (CFAN). The participants were from families carrying mutations in known causative genes of FAD: amyloid precursor protein (APP), presenilin 1 (PSEN1), or presenilin 2 (PSEN2). In this study, mutation carriers with an estimated years to symptom onset of 8-10 years were included and determined to have preclinical AD. Age- and sex-matched mutation non-carriers within the families served as normal controls.
Ethics oversight	This study was approved by the Institutional Review Board of Xuanwu Hospital, Capital Medical University.

Note that full information on the approval of the study protocol must also be provided in the manuscript.

Field-specific reporting

Please select the one below that is the best fit for your research. If you are not sure, read the appropriate sections before making your selection.

☒ Life sciences ☐ Behavioural & social sciences ☐ Ecological, evolutionary & environmental sciences

For a reference copy of the document with all sections, see nature.com/documents/nr-reporting-summary-flat.pdf

Life sciences study design

All studies must disclose on these points even when the disclosure is negative.

Sample size	The sample size was decided according to a previous paper(DOI: 10.1016/j.jalz.2015.12.012), which included 77 controls and 103 AD. Our study recruited a longitudinal cohort (Cohort 1) , including 126 preclinical AD, and 123 controls. Cohort 2 included 51 carriers of an APP/PS mutation and 52 non-carriers. We believe that our sample size is enough for statistic analysis and conclusion.
Data exclusions	No data were excluded from the analyses.
Replication	The replication cohort successfully replicated the main findings from Cohort 1.
Randomization	The allocation of the participants was not randomized. We grouped the participants according to their diagnosis. The sex and age of the participants between controls and preclinical AD were matched.
Blinding	The investigators were blinded to group allocation during data collection and analysis.

Reporting for specific materials, systems and methods

We require information from authors about some types of materials, experimental systems and methods used in many studies. Here, indicate whether each material, system or method listed is relevant to your study. If you are not sure if a list item applies to your research, read the appropriate section before selecting a response.

Materials & experimental systems

n/a	Involved in the study
☐	☒ Antibodies
☒	☐ Eukaryotic cell lines
☒	☐ Palaeontology and archaeology
☒	☐ Animals and other organisms
☒	☐ Clinical data
☒	☐ Dual use research of concern
☒	☐ Plants

Methods

n/a	Involved in the study
☒	☐ ChIP-seq
☒	☐ Flow cytometry
☒	☐ MRI-based neuroimaging

Antibodies

Antibodies used

Commercially available kit (Quanterix Simoa) for Plasma Aβ42, Aβ40, P-tau181, T-tau, and NfL:cat#101995 for Aβ42, Aβ40 and T-tau;cat#103714 for P-tau181 and cat#103186 for NfL.
Commercially available kit (INNOTEST ELISA) for CSF Aβ42(cat#81576), Aβ40(cat#81585), P-tau181(cat#81581), T-tau(cat#81572), and NfL(cat#20-8002).

Validation

Validation details can be found at the manufacturer's website.
